# Vancomycin AUC_24_ Estimation for Children with Bayesian Forecasting and First-Order Equations to Update Dose Recommendations

**DOI:** 10.3390/antibiotics15090852

**Published:** 2026-09-01

**Authors:** Rosa Kalliainen, Marjo Renko, Ulla Sankilampi, Merja Kokki, Veli-Pekka Ranta

**Affiliations:** 1 Department of Pediatrics, Kuopio University Hospital, FI-70210 Kuopio, Finland; rosakall@student.uef.fi (R.K.); marjo.renko@uef.fi (M.R.); ulla.sankilampi@pshyvinvointialue.fi (U.S.); 2 Institute of Clinical Medicine, University of Eastern Finland, FI-70210 Kuopio, Finland; merja.kokki@pshyvinvointialue.fi; 3 Department of Anesthesiology and Intensive Care, Kuopio University Hospital, FI-70210 Kuopio, Finland; 4 School of Pharmacy, University of Eastern Finland, FI-70210 Kuopio, Finland

**Keywords:** vancomycin monitoring, pharmacokinetics, area under the curve, pediatrics

## Abstract

**Objectives:** This retrospective study aimed to estimate vancomycin area under the curve over 24 h (AUC_24_) at steady state for pediatric patients from 1 month to 18 years to update the dose recommendations in a tertiary hospital. **Methods**: A total of 46 vancomycin treatments from 42 patients in the years 2014–2024 were analyzed. AUC_24_ was estimated for the initial dose regimen using Bayesian forecasting and a trough concentration (n = 32) or first-order pharmacokinetic (PK) equations and a pair of practical peak and trough concentrations at steady state (n = 14). Three published population PK models (Colin, Kloprogge, and Le models) were compared using typical parameter values (a priori predictions) to find a suitable model for Bayesian estimation. For the Le model, the effect of converting plasma creatinine (PCr) measured with IDMS-traceable enzymatic assay to Jaffe method value was evaluated using published equations. **Results**: Le model without enzymatic-to-Jaffe PCr conversion and the Colin model were suitable for Bayesian forecasting. These models gave similar Bayesian AUC_24_ estimates (a posteriori). In all 46 treatments, estimated AUC_24_ was within the currently recommended range of 400–600 mg·h/L in 8 (17%) treatments, below this range in 36 (78%) treatments, and above this range in 2 (4%) treatments, respectively. Estimated AUC_24_ was below 300 mg·h/L in 46% of treatments. **Conclusions**: Le and Colin models were suitable for our pediatric patients for Bayesian forecasting. AUC_24_ estimates for the initial dose regimen were lower than commonly recommended. A more uniform dosing policy is needed in our hospital, and higher initial doses should be considered.

## 1. Introduction

Vancomycin is used to treat severe staphylococcal and enterococcal infections. The therapeutic range for vancomycin exposure is narrow to assure efficacy and avoid nephrotoxicity, and therapeutic drug monitoring (TDM) is needed. The USA 2020 consensus guideline recommended the same exposure target in severe methicillin-resistant *Staphylococcal aureus* (MRSA) infections for both adults and children: area under the curve over 24 h (AUC_24_) of 400 to 600 mg·h/L, assuming vancomycin minimum inhibitory concentration (MIC) of ≤1 mg/L for MRSA [1]. The same AUC_24_ target range has been used to guide vancomycin dosing in methicillin-sensitive *S. aureus* and coagulase-negative staphylococcal infections, as well as in enterococcal infections.

The recommended and most used AUC_24_ estimation methods for children receiving intermittent vancomycin infusions are Bayesian forecasting using one or two concentrations and the application of first-order pharmacokinetic (PK) equations with a pair of practical peak and trough concentrations measured at steady state [1,2].

Numerous population PK models of vancomycin are available for neonatal and pediatric patients for use in Bayesian forecasting [2,3,4]. The aim in model selection is that the vancomycin PK of target patients resembles that of patients used in the model building. In practice, the suitability of a published PK model can be evaluated by comparing the model-predicted concentrations obtained with the typical model parameters (a priori predictions) with the observed concentrations from the target patients [5,6].

Even though AUC_24_ monitoring is currently recommended, trough monitoring is still applied in many hospitals because of the costs of commercial Bayesian software or limited training in PK modelling. Trough monitoring is supported by the data on the correlation between AUC_24_ and trough concentration. A common trough target range for pediatric patients is 10–15 mg/L. With the dose intervals of 6 h and 8 h, this trough range corresponds to an AUC_24_ range of 400–600 mg·h/L, but only roughly, since between-subject variability is large and an AUC_24_ of 400 mg·h/L has been reached with trough concentrations below 10 mg/L [7,8,9].

This study belongs to a larger retrospective survey to update vancomycin dose recommendations for children in Kuopio University Hospital. In our previous study on neonates and small infants, we found that an increased daily dose was needed for preterm neonates with postmenstrual age (PMA) < 29 weeks to ensure an adequate AUC_24_ [10].

In the current retrospective study, a wider age range is covered, from PMA of 44 weeks (corrected age of 1 month) up to the age of 18 years. A plain trough concentration was measured for most children, and AUC_24_ was estimated with Bayesian forecasting. Three published population PK models were compared to find a suitable model for Bayesian estimation. For one-third of the children, a pair of practical peak and trough concentrations was measured at steady state, and AUC_24_ was calculated with first-order PK equations. The main aim was to estimate AUC_24_ for the initial dose regimen to assess and revise the dose recommendations in our hospital.

## 2. Results

### 2.1. Patients, Vancomycin Dosing, and Measured Concentrations

The number of pediatric patients (PMA ≥ 44 weeks and age < 18 years) with at least one vancomycin trough concentration in our hospital database during 2014–2024 was 52. Ten patients were excluded because of missing vancomycin dosing data or severe kidney disease. The final data consisted of 46 vancomycin treatments in 42 patients.

The patients with Bayesian AUC_24_ estimation were divided into Groups 1 and 2 based on their age (below or above 12 years). The patients with AUC_24_ estimation by first-order PK equations formed Group 3, and this group contained most of the youngest children with median (interquartile range (IQR)) PMA of 55 (46; 72) weeks. The patient characteristics are shown in Table 1.

The most common bacterial isolates were *S. aureus* (MRSA 8% of *S. aureus* isolates), *S. epidermidis* and *E. faecalis* (Table 1). In blood and cerebrospinal fluid, the ranges of vancomycin MIC values measured with a gradient strip for methicillin-sensitive *S. aureus*, MRSA, and *S. epidermidis* were 0.5–0.75 mg/L, 0.5–1.5 mg/L, and 0.75–2 mg/L, respectively.

The initial daily dose in the oldest children (Group 2) was lower than in other groups, whereas the measured trough concentrations were similar in all groups (Table 2).

### 2.2. Part 1: Model Comparison

#### 2.2.1. PK Model Comparison for Typical Virtual Finnish Boys

**Figure 1:**
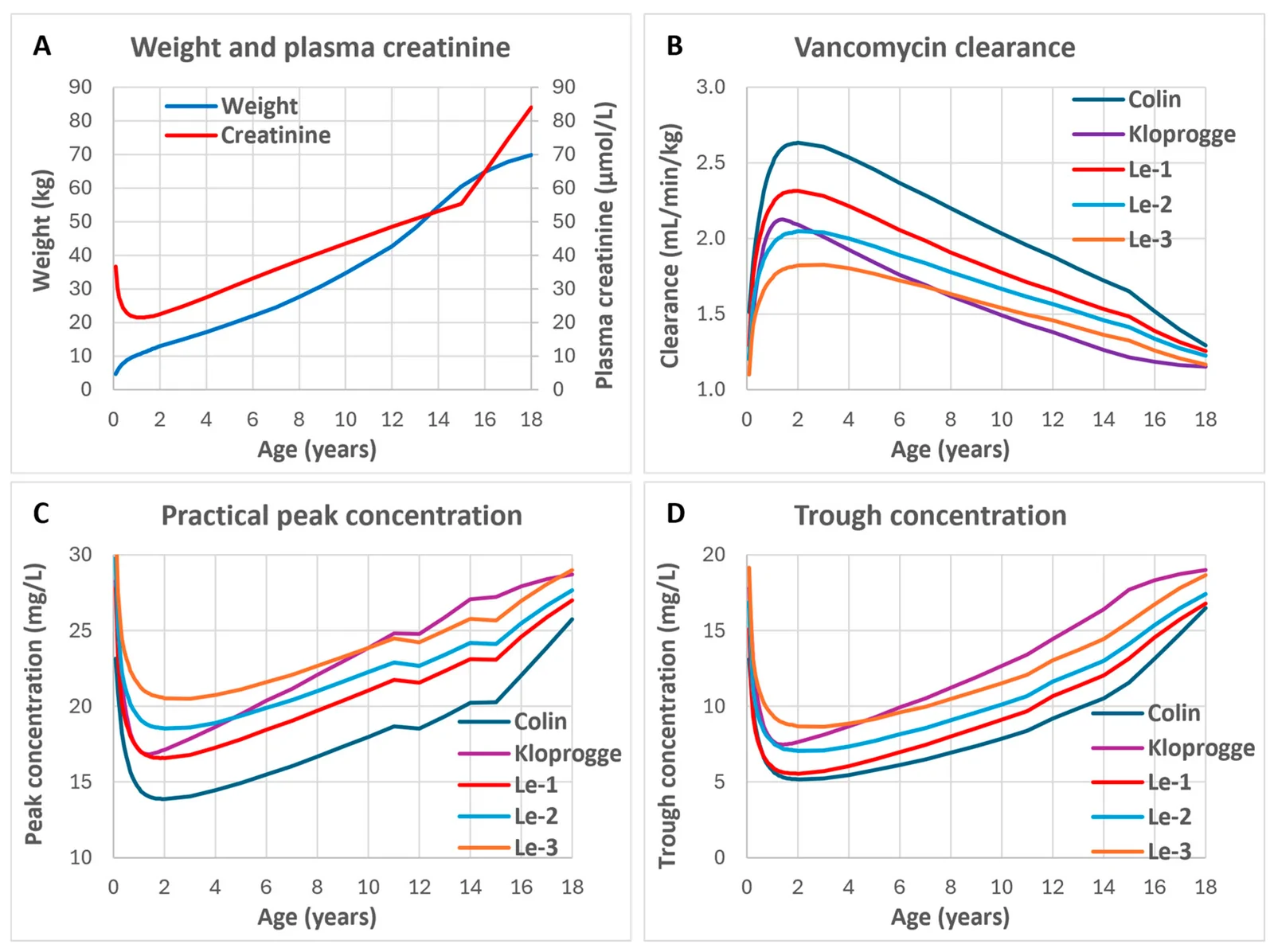
Comparison of five pharmacokinetic models for typical virtual Finnish boys (1 month–18 years) with vancomycin dose of 15 mg/kg every 8th hour at steady state. (**A**) Weight and plasma creatinine of virtual patients, (**B**) vancomycin clearance, (**C**) practical peak concentration at two hours after the end of infusion, and (**D**) trough concentration at the end of dose interval.

PK calculations for typical virtual Finnish boys revealed large differences between the published population PK models (Figure 1). Vancomycin clearance (CL) was the highest, and the steady-state concentrations were the lowest, with the Colin and Le-1 models, whereas the Kloprogge and Le-3 models were at the opposite end, respectively.

#### 2.2.2. PK Model Comparison and Model Selection for Patients in Groups 1 and 2

Trough concentrations were predicted with typical parameter values (a priori predictions) with five population PK models for 28 of 32 treatments in Groups 1 and 2. In these treatments, trough concentration was measured at steady state (after at least six elimination half-lives estimated with the Le-1 model), allowing the use of steady-state equations.

The model-predicted concentrations obtained with the Colin and Le-1 models were the lowest among all models, and these predictions were closest to the observed concentrations (Table 3). The root mean square error (RMSE) of the Colin model (3.73 mg/L was lower than that of the Le-1 model (4.56 mg/L). The performance of the Colin model and Le-1 model was adequate even though both models tended to overpredict the concentrations. At the extreme, there were 6 (21%) and 7 (25%) percent errors above +100% with the Colin and Le-1 models, respectively. In four of these cases, the measured concentration was below 3 mg/L (below the lower limit of quantification, LOQ), and it was set to 1.5 mg/L for pharmacokinetic calculations. Therefore, the calculated percent error was a rough estimate in these cases.

Two enzymatic-to-Jaffe conversions of plasma creatinine (PCr) in the Le-2- and Le-3 models increased the mean and median percent errors and RMSE markedly when compared to the Le-1 model with no conversion (Table 3).

Colin and Le-1 models were chosen for Bayesian forecasting.

#### 2.2.3. Bayesian Forecasting with Le-1 and Colin Models for Patients in Groups 1 and 2

Bayesian AUC_24_ and CL estimates obtained with the Le-1 and Colin models were similar when using both the reduced and original residual error models (Table 4 and Table 5). The Le-1 model with the reduced error model was used as the reference model since its features (one-compartment model and the lowest prediction errors) are most similar to the first-order equation method that was used for Group 3.

As expected, the prediction errors and percent errors obtained with the original error models were higher than with the reduced error models. For example, seven percent errors between +36% and +112% were obtained with the Colin original error model in the combined Groups 2 and 3, and in these cases AUC_24_ estimates were 19–46% higher than by the Colin reduced error model, respectively (see the footnote comparison in Table 4 and Table 5). In cases with the five highest percent errors, the trough concentration was below LOQ and was set to one half of LOQ, meaning that the AUC_24_ estimate was rough with all models.

**Figure 2:**
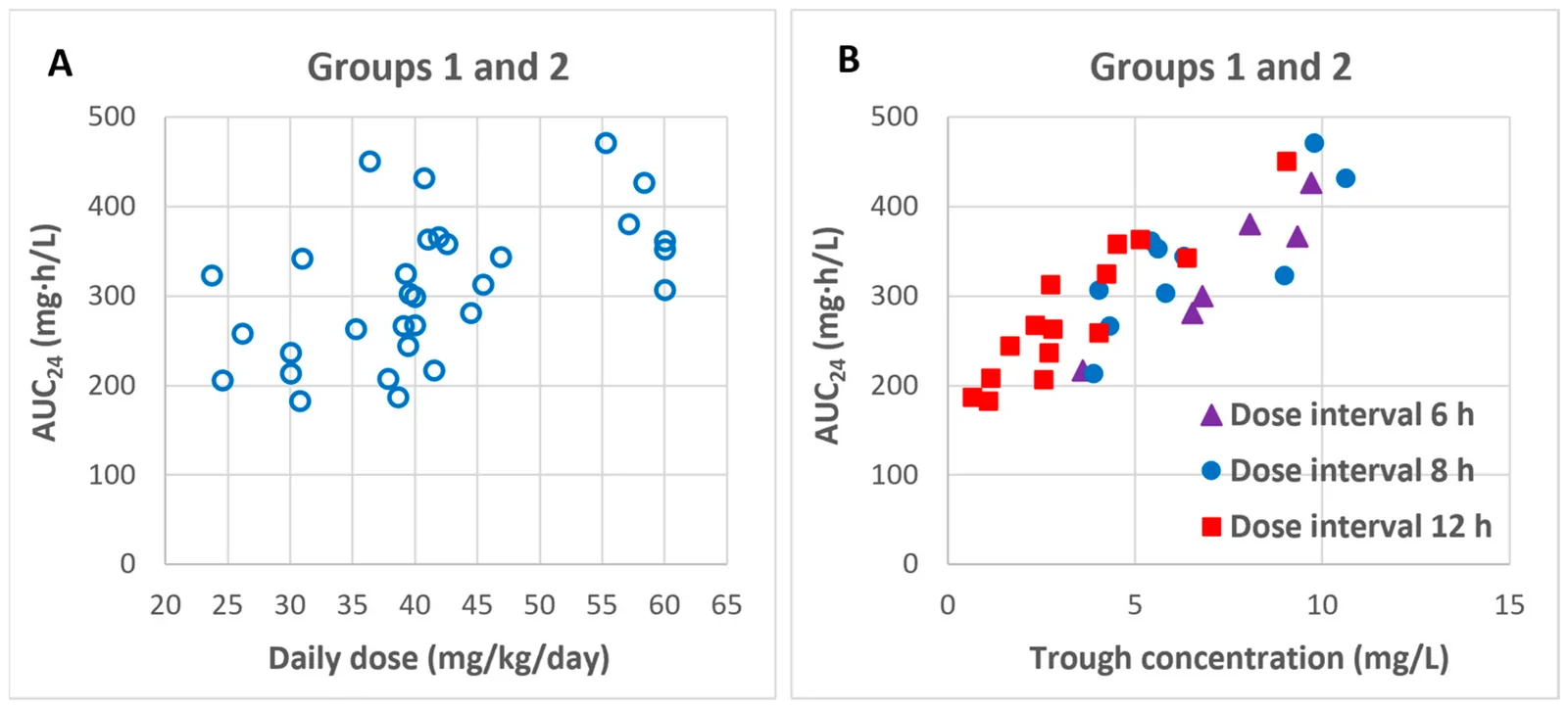
Relationship between (**A**) vancomycin AUC_24_ and initial daily dose, and (**B**) AUC_24_ and trough concentration for children from 0.3 to 17 years in Groups 1 and 2. Steady-state AUC_24_ and trough concentration at the end of dose interval were estimated with Bayesian forecasting using Le-1 model and the reduced residual error model.

Estimated AUC_24_ generally increased with the daily dose, and the correlation between estimated AUC_24_ and trough at the end of dose interval at steady state obtained with the Le-1 reduced error model suggested that a trough concentration around 10 mg/L corresponded to AUC_24_ of 400 mg·h/L (Figure 2).

Median CL obtained with different models was 2.43–2.63 mL/min/kg in Group 1 and 1.83–2.02 mL/min/kg in Group 2, respectively (Table 4 and Table 5). Median apparent volumes of distribution (Vd, V1 and V2) in Groups 1 and 2 were 0.58–0.65 L/kg (Table 4 and Table 5), which were close to the typical values in the Le and Colin models (Table S1).

The steady-state concentration curves obtained with the Le-1 and Colin models for the median children in Groups 1 and 2 were similar, except that the terminal elimination half-life after stopping vancomycin treatment was much longer with the Colin model (Table 4 and Table 5; Figure S1).

#### 2.2.4. Group 3 with AUC_24_ Estimation by First-Order PK Equations and Bayesian Forecasting

A pair of trough and practical peak concentrations was measured at steady state for patients in Group 3, and PK parameters were estimated with first-order equations. Median (min-max) parameters were the following:

- AUC_24_ at steady state 349 (211–640) mg·h/L;
- CL 2.17 (1.01–3.15) mL/min/kg;
- Vd 0.63 (0.34–0.95) L/kg;
- Elimination half-life 3.6 (1.7–7.5) h.

The time interval from the start of vancomycin therapy to the trough measurement (measured before the peak) was 6–7 estimated elimination half-lives in two treatments and over 10 half-lives in other treatments.

The differences in AUC_24_ and CL estimates between the first-order equation method and Bayesian forecasting with the Le-1 and Colin models were relatively small (Table S4).

### 2.3. Part 2: Summary of AUC_24_ and CL Estimates for Three Age Groups

A data summary for three age groups was compiled using the parameter estimates obtained with first-order equations and the Le-1 reduced residual error model (Table 6). Other estimation methods would have given similar results since the differences in the parameter estimates were small (Table 4, Table 5 and Table S4).

Estimated AUC_24_ in different age groups was within the currently recommended range of 400–600 mg·h/L in 11–29% of treatments (Table 6). In all 46 treatments, estimated AUC_24_ was within 400–600 mg·h/L in 8 (17%) treatments, below this range in 36 (78%) treatments, and above this range in 2 (4%) treatments, respectively. Estimated AUC_24_ was below 300 mg·h/L in 21 (46%) treatments.

The difference between median initial daily dose and the calculated dose to obtain median AUC_24_ of 400 mg·h/L was particularly large for the children from 1 to 12 years (41 versus 60 mg/kg/day) and from 12.1 to 17 years (31 versus 48 mg/kg/day) (Table 6). The dose was increased most often in the oldest age group (46% of treatments). In this group, the lowest initial doses of 24–31 mg/kg/day (lower than or equal to the median dose) were used in seven treatments, and the dose was increased during six of these treatments.

The removal of the second treatment in two patients and the second and third treatments in one patient (there were 46 treatments in 42 patients) and the practice of setting below-LOQ concentrations to LOQ/2 had only a minor effect on median (min–max) CL and AUC_24_ values (Table S5).

### 2.4. Renal Function and Mortality

The monitoring of PCr was variable and often sparse. PCr was measured both before and after the vancomycin treatment in eight patients. PCr declined in five patients by 2–38%, remained the same in one patient, and increased in two patients by 9% and 19%, respectively. None of the patients died within 15 days from the end of vancomycin treatment.

## 3. Discussion

The aim of this study was to estimate vancomycin AUC_24_ obtained with the initial dose regimen for pediatric patients from 1 month to 18 years to update the dose recommendations in our hospital. The study was retrospective and covered the years 2014–2024. Due to our hospital’s strict policy on using vancomycin, the data were limited: 46 treatments from 42 different patients were analyzed.

The recommended AUC_24_ for serious MRSA infections in the USA 2020 consensus guideline is 400–600 mg·h/L, assuming a vancomycin MIC of 1 mg/L measured with broth microdilution [1]. This target range is also used to guide vancomycin dosing in other staphylococcal infections and enterococcal infections. A recent systematic review on vancomycin effectiveness in pediatric and neonatal staphylococcal infections concluded that there are limited pediatric data on AUC_24_ or AUC_24_/MIC thresholds and further research is needed [11].

Vancomycin exposure targets for children are evaluated continuously by studying the efficacy and the incidence of acute kidney injury [12,13,14]. In a recent study in 1714 children from 1 month to 19 years, the mortality within 72 h of the last vancomycin dose was low (1.9%) even though only 43% of patients achieved an AUC_24_ of 400 mg·h/L [14]. Median (IQR) daily dose and AUC_24_ obtained with Bayesian forecasting were 50 (42; 57) mg/kg/day and 372 (292; 487) mg·h/L, respectively. The authors concluded that the clinical necessity for increased doses remains questionable based on the low mortality, but mentioned that increased doses might reduce the duration of infection. The time to bacteremia clearance has been suggested as an important outcome measure for future research since the risk of complications increases with each additional day of bacteremia [11].

In our study, mortality was zero within 15 days from the end of vancomycin treatment. The most common bacterial isolates were *S. aureus* (MRSA 8% of *S. aureus* isolates), *S. epidermidis*, and *E. faecalis*. Median initial daily doses in the age ranges of 1–12 months, 1–12 years, and 12.1–17 years were 44, 41, and 31 mg/kg/day, and median AUC_24_ was 360, 300, and 323 mg·h/L, respectively (Table 6). Estimated AUC_24_ was between 400 and 600 mg·h/L in 17% (8/46) of all treatments, and below this range in 78% (36/46) of treatments.

Based on median CL for each age group, the calculated daily doses of 48, 60, and 48 mg/kg/day would be needed to obtain a median AUC_24_ of 400 mg·h/L (Table 6). If the aim is that a large majority of patients will reach an AUC_24_ of 400 mg·h/L with the initial dose regimen, the doses need to be higher, in practice around 60 to 70 mg/kg/day. As examples, the following vancomycin doses are recommended for children with normal kidney function: USA consensus for serious MRSA infection [1]: 60–80 mg/kg/day (3 months to <12 years) and 60–70 mg/kg/day (≥12 years); USA Red Book (≥1 month) [15]: 45–60 mg/kg/day and up to 60–70 mg/kg/day in invasive MRSA; Kinderformularium in Netherlands (≥1 month) [16]: 60 mg/kg/day. The recommended maximum dose is 3600 or 4000 mg/day [1,16].

The exact AUC_24_ target for the basis of our future dose recommendations for children with normal kidney function has yet to be decided. However, the marked difference between the median initial dose and the calculated dose to obtain a median AUC_24_ of 400 mg·h/L in the two oldest age groups (41 versus 60 mg/kg/day; 31 versus 48 mg/kg/day) (Table 6) will be taken into consideration. The frequency of dose increases will also receive attention. The clinicians decided dose changes based on the trough concentration (AUC_24_ not available) and the normal monitoring of a patient’s clinical status. In children from 12.1 to 17 years, the lowest initial doses of 24–31 mg/kg/day (lower than or equal to the median dose) were given to seven patients, and the dose was increased in six patients to the median dose of 46 mg/kg/day in 1–3 steps (Table 6). This suggests that doses ≤31 mg/kg/day are too low for this age group. The highest initial doses in our study were 55–60 mg/kg/day (six patients > 1 year), and 60 mg/kg/day will be one of the recommended initial doses.

Although AUC_24_ monitoring is currently recommended, trough monitoring will be continued in our hospital for practical and economic reasons. In the current study, median observed trough concentration was 6 mg/L in all groups, and 62–86% of the trough concentrations were measured within 1 h of the end of dose interval (Table 2). Published pediatric studies indicate that the common trough target range of 10–15 mg/L only roughly corresponds to an AUC_24_ range of 400–600 mg·h/L [7,8,9]. The trough concentrations above 15 mg/L should normally be avoided due to an increased risk of nephrotoxicity [1,17]. More flexibility might be allowed at the lower limit, since an AUC_24_ of 400 mg·h/L has been reached with trough concentrations below 10 mg/L (e.g., 7–9 mg/L), and a patient’s clinical response is more important than the exact trough concentration. For consistent results, the trough concentration needs to be measured within 1 h or preferably within 0.5 h of the end of dose interval since the elimination half-lives obtained with the Le-1 model (Table 4 and Table 5) and first-order equations (Section 2.2.4) were short, with a median of around 3–4 h and the lowest values around 2 h. Furthermore, a dose interval of 12 h is too long for children with normal kidney function. Seven of nine below-LOQ concentrations occurred with this dose interval.

Both Bayesian forecasting and first-order PK equations (Sawchuk-Zaske method [18]) were used to estimate AUC_24_ for our patients. First-order PK equations require the measurement of a pair of practical peak and trough concentrations at steady state. Bayesian estimation is more flexible since it is applicable also with one measured concentration and a steady state is not required [2].

Three published population PK models were compared to empirically determine a suitable model for our patients for Bayesian forecasting. The Colin [19] and Kloprogge [20] models were tested since they performed well in a previous model comparison [5]. The Le model [7] was included since it has been used in several studies [2]. The published results obtained with these models suggested that typical vancomycin exposure for the same patient will differ between the models, and this was confirmed in our model comparison with the virtual Finnish boys (Figure 1).

The model-predicted trough concentrations obtained with typical parameter values (a priori predictions) were closest to the observations with the Colin and Le models (Table 3). Both models were chosen for Bayesian forecasting. Our version of the Le model (called the Le-1 model) differed from the original model since PCr was measured in our study with an IDMS-traceable enzymatic assay (IDMS-Enz), whereas the Jaffe method was used in the original study [7]. Jaffe values are generally higher than IDMS-Enz values. We tested two published equations [21] to convert IDMS-Enz values to Jaffe values in the Le-2 and Le-3 models, but a priori mean and median percent errors and RMSE increased significantly. The best performance of the Le model without any Jaffe conversion suggests that vancomycin PK in our patients differed to some extent from that of the patients in Le et al. [7].

Bayesian AUC_24_ and CL estimates (a posteriori) obtained with the Le-1 and Colin models were similar when using both the reduced and original residual error models (Table 4 and Table 5). In the reduced error model, the proportional residual error was set to 15% of the measured concentration to force the model-predicted concentrations closer to the observations [22,23]. The prediction error decreased since the trade-off feature of Bayesian forecasting, the balancing of prior data (population model) and posterior data (measured concentration, was adjusted in favour of posterior data. We tested the reduced error model on an empirical basis in our retrospective survey, whereas its pros and cons need to be evaluated carefully in actual patient care.

For most of our patients, a single trough concentration was used in Bayesian forecasting. In a previous study with a pediatric one-compartment model of vancomycin, a pair of near-peak and trough concentrations provided more accurate and precise Bayesian AUC_24_ estimates than a plain trough concentration with an 8% difference in median AUC_24_ estimates [24]. However, Bayesian fitting of a single concentration is an acceptable method for vancomycin TDM. We consider that our Bayesian AUC_24_ estimates are accurate enough for the purpose of our study, especially because the Le-1 and Colin models gave similar results.

A limitation in our study was the small number of patients and treatments in relation to the wide age range from 1 month to 18 years. The focus was on pharmacokinetics, whereas the evaluation of efficacy was limited to mortality. Nephrotoxicity was not studied. The monitoring of PCr levels was variable and often sparse, and the published criteria for acute kidney injury based on the changes in PCr could not be used. The effect of augmented renal clearance on vancomycin exposure was not addressed since the estimated glomerular filtration rate (eGFR) was not available. Due to these limitations and the exploratory and descriptive nature of the study, the dose recommendations cannot be based solely on this study, but published guidelines and scientific literature must be consulted.

In conclusion, the Le model was suitable for our pediatric patients for Bayesian forecasting without converting IDMS-traceable enzymatic plasma creatinine to the Jaffe method value. The Colin model gave similar AUC_24_ estimates. AUC_24_ estimation with Bayesian fitting and first-order PK equations showed that the initial daily doses in our hospital provided lower vancomycin exposure than commonly recommended. A more uniform dosing policy is required in our hospital, and higher initial doses should be considered. The guidance should also give detailed advice on the monitoring of vancomycin levels and kidney function.

## 4. Materials and Methods

### 4.1. Patients and Their Grouping

Our retrospective study was approved by the medical director of Kuopio University Hospital (No 114/2019). The inclusion criteria for the children treated with vancomycin in all pediatric wards of Kuopio University Hospital during 2014–2024 were as follows. (1) Postmenstrual age (PMA) ≥ 44 weeks (corrected age ≥1 month) and postnatal age <18 years. (2) At least one vancomycin trough concentration was measured. (3) Vancomycin dosing data and necessary demographic data were available. (4) The patient did not have severe kidney dysfunction or kidney disease. The same patient was included more than once if the vancomycin treatments were given at different age ranges: (1) 1–3 months, (2) 3–12 months, (3) 1–6 years, (4) 6–12 years, and (5) 12–17.9 years.

The first part of the study was a methodological model comparison. For this purpose, the patients and treatments were grouped primarily based on the available concentration data and AUC_24_ estimation method (see Section 4.4 and Section 4.5) with an additional age limit at 12 years: Group 1: Bayesian estimation of a single trough concentration for patients below 12 years; Group 2: Bayesian estimation of a single trough concentration for patients above 12 years; and Group 3: First-order PK equations using a pair of peak and trough concentrations for small children treated with the protocol by the neonatal intensive care unit (NICU). The second part of the study was the evaluation of AUC_24_ values obtained with the initial dose regimen and the most suitable AUC_24_ estimation methods for the basis of revised dose recommendations for three age groups: 1–12 months, 1–12 years, and 12.1–17.9 years.

### 4.2. Dosing and Monitoring of Vancomycin

For the infants treated at NICU, the recommended vancomycin dose in the unit’s guidance (PMA > 36 weeks) was 15 mg/kg every 8th hour as 1 h intravenous infusion. The trough concentration was measured after two or three days, and the practical peak concentration 2 h after the end of the next infusion. The target concentrations were 5–15 mg/L (trough) and 25–40 mg/L (practical peak). The NICU guidance was also used for several small children treated at the general pediatric ward. For other children, the initial dose regimen was chosen by the treating clinicians, and the trough concentration was measured typically after one or two days. None of the patients received a loading dose.

### 4.3. Bioassays

Serum vancomycin was measured using enzyme-multiplied immunoassay technique (EMIT) with a limit of quantification (LOQ) of 2 mg/L until Autumn 2017 and thereafter with immunoassay based on kinetic interaction of microparticles in solution (KIMS) with LOQ of 3 mg/L. Plasma creatinine (PCr) was determined with an isotope dilution mass spectrometry traceable enzymatic (IDMS-Enz) assay. These bioassays were performed with cobas 6000 and 8000 c-modules (Roche Diagnostics, Basel, Switzerland) in an accredited (ISO 15189:2022: [25]) clinical chemistry laboratory (ISLAB, Kuopio, Finland).

### 4.4. Pharmacokinetic Analysis with Bayesian Forecasting

When plain trough concentration was available for the initial dose regimen, pharmacokinetic analysis was performed with Bayesian forecasting (Groups 1 and 2). The steps in the protocol were (1) PK model comparison for virtual patients to evaluate the differences between published population PK models, (2) PK model comparison for patients in Groups 1 and 2 using typical parameter values (a priori prediction) to select suitable models for Bayesian estimation, and (3) Bayesian forecasting with suitable models for patients in Groups 1 and 2 to estimate PK parameters and AUC_24_ (a posteriori prediction).

#### 4.4.1. PK Model Comparison for Typical Virtual Finnish Boys

Three population PK models for vancomycin were compared, including three different versions of one of the models (model parameters in Table S1): (1) Colin et al. 2019 [19]: two-compartment model developed for all age groups; (2) Kloprogge et al. 2019 [20]: two-compartment model for neonates and pediatric patients; and (3) Le et al. 2013 [7]: one-compartment model for pediatric patients over 3 months. Three different versions of the Le model were evaluated: (A) Le-1: PCr measured with IDMS-Enz assay was used to calculate vancomycin clearance (CL); (B) Le-2: Measured IDMS-Enz PCr was converted to Jaffe method value using the enzymatic-to-Jaffe equation [21] for CL calculation; and (C) Le-3: Measured IDMS-Enz PCr was converted to Jaffe method value using the IDMS-Enz-to-Jaffe equation [21] for CL calculation.

Typical virtual Finnish boys (1 month to 18 years) were created by taking the mean weight from Finnish growth data [26] and mean age-adjusted PCr from international compilations [27,28]. Vancomycin CL, steady-state trough concentration, and practical peak concentration at 2 h after the end of infusion were calculated for a dose of 15 mg/kg every 8th hour. The duration of infusion was 1 h for doses up to 600 mg, 1.5 h for doses up to 900 mg, and 2 h for doses above 900 mg, respectively.

#### 4.4.2. PK Model Comparison and Model Selection for Patients in Groups 1 and 2

Trough concentration at the recorded time after the start of previous infusion was predicted with typical parameter values (a priori prediction) with five population PK models (Section 4.4.1) for those patients in Groups 1 and 2 for whom the trough concentration was measured at steady state. This allowed the use of steady-state equations. The IDMS-Enz PCr value for the calculation of vancomycin CL was defined based on the availability of PCr measurements (Appendix A).

The prediction error, percent error, and root mean square error (RMSE) were calculated for the trough concentration (conc) as

Prediction error (mg/L) = Predicted conc − Measured conc(1)

Percent error (%) = 100 · (Predicted conc − Measured conc)/Measured conc(2)

(3) $$\mathrm{RMSE}\;\left(\mathrm{mg}/L\right)=\sqrt{\frac{\sum_{i=1}^{n}{\left(\mathrm{Prediction}\;\mathrm{error}\right)}^{2}}{n}}$$

Suitable models were selected based on mean and median percent errors, RMSE, fraction of predictions within 15% or 2.5 mg/L of the measured values [6], and simplicity of the PK model.

#### 4.4.3. Bayesian Forecasting with the Most Suitable Models for Patients in Groups 1 and 2

Bayesian forecasting of PK parameters (a posteriori prediction) was made with LibreOffice 7.4 (LibreOffice Community) using the most suitable models, full dosing data (without steady state assumption), and the first trough concentration (a detailed description in Table S2). Before vancomycin fittings, the correctness of Bayesian parameter estimation was validated (Table S3).

Two residual error models were compared in the parameter estimation: (1) the published original error model, and (2) the reduced error model where the coefficient of variation (CV%) of proportional residual error was 15% of the measured concentration with normal distribution (Table S2). The reduced error was used as a practical way to improve the model fit, and CV% of 15% was based on the typical assay precision [22,23].

When the measured vancomycin trough concentration was below LOQ, it was set to one half of LOQ. The IDMS-Enz PCr value for the calculation of vancomycin CL was defined based on the availability of SCr measurements (Appendix A).

Steady-state AUC_24_ and the daily dose to obtain an AUC_24_ of 400 mg·h/L were calculated using Equations (4) and (5):

Steady-state AUC_24_ (mg·h/L) = Total daily dose (mg)/CL (L/h) (4)

Dose for AUC_24_ of 400 mg·h/L (mg/kg/day) = CL (L/h/kg) × 400 (mg·h/L/day) (5)

### 4.5. Pharmacokinetic Analysis with First-Order PK Equations

When a pair of trough and practical peak concentrations were measured at steady state (NICU protocol for Group 3), pharmacokinetic analysis was performed with first-order PK equations for a one-compartment model (Sawchuk-Zaske method [18]) as described previously [23]. When the measured trough concentration was below LOQ, it was set to one half of LOQ.

### 4.6. Statistical Analysis

Descriptive statistics were used. Data were described as median (lower quartile; upper quartile) or median (minimum–maximum) or n (% of treatments).

## Figures and Tables

**Table 1:** Patient characteristics.

Characteristic	Group 1 Age 0.3–12 Years Bayesian Estimation (n = 19)	Group 2 Age 12.1–17 Years Bayesian Estimation (n = 13)	Group 3 Age 1–15 Months First-Order Equations (n = 14)
Girls/boys	7/12	5/8	3/11
Age (years)	3.4 (1.8; 8.9)	14 (13; 16)	0.35 (0.19; 0.60)
Weight (kg)	17 (11; 34)	65 (40; 76)	4.8 (3.7; 6.4)
Plasma creatinine (µmol/L) ^a^	24 (21; 36)	47 (32; 61)	21 (18; 25)
Treatment in neonatal or general ICU	2 (11%)	2 (15%)	10 (71%)
Clinical specialty			
Oncology	6 (32%)	4 (31%)	3 (21%)
Surgery	1 (5%)	4 (31%)	3 (21%)
Neurosurgery	5 (26%)	2 (15%)	2 (14%)
Pediatrics	7 (37%)	2 (15%)	6 (43%)
Other	0	1 (8%)	0
Central venous catheter	10 (53%)	5 (38%)	7 (50%)
Positive blood or cerebrospinal culture ^b^	8 (42%)	2 (15%)	3 (21%)
*Staphylococcus aureus*	5	0	3
*Staphylococcus epidermidis*	6	2	0
Positive culture of other tissue samples ^b^	6 (32%)	8 (62%)	9 (64%)
*Candida albicans*	0	0	3
*Enterococcus faecalis*	1	3	3
*Staphylococcus aureus*	4	2	9
*Staphylococcus epidermidis*	0	3	2
Concomitant drugs ^c^			
Piperacillin/Tazobactam	5 (26%)	7 (54%)	1 (7%)
Cefuroxime or Ceftriaxone	10 (53%)	4 (31%)	6 (43%)
Meropenem	4 (21%)	3 (23%)	6 (43%)
Tobramycin	2 (11%)	3 (23%)	3 (21%)
Metronidazole	1 (5%)	3 (23%)	3 (21%)
Trimethoprim/Sulfadiazine	6 (32%)	4 (30%)	3 (21%)
Amphotericin B	0	1 (8%)	3 (21%)
Fluconazole	5 (26%)	2 (15%)	3 (21%)
Furosemide	1 (5%)	2 (15%)	6 (43%)
Spironolactone	0	0	3 (21%)
Ibuprofen or Naproxen	9 (47%)	6 (46%)	3 (21%)
Contrast agent before vancomycin course	9 (47%)	5 (38%)	2 (14%)
Concomitant contrast agent	4 (21%)	2 (15%)	2 (14%)

Data are presented as median (lower quartile; upper quartile) or n (% of treatments). There were 46 treatments in 42 patients. ^a^ n = 17 for Group 1 and n = 13 for Group 3. Of all 43 values, 39 were pre-treatment values and 4 were the first value during the treatment. ^b^ Most common isolates are reported. The percentage of MRSA from all *S. aureus* isolates was 8%. ^c^ Antibacterial and antifungal agents, diuretics and non-steroidal anti-inflammatory agents are reported.

**Table 2:** Vancomycin dosing and measured concentrations.

Variable	Group 1 Age 0.3–12 Years Bayesian Estimation (n = 19)	Group 2 Age 12.1–17 Years Bayesian Estimation (n = 13)	Group 3 Age 1–15 Months First Order Equations (n = 14)
Initial daily dose (mg/kg/day)	41 (40; 46)	31 (30; 39)	44 (42; 45)
Initial dose interval (h)	8 (6; 12)	12 (8; 12)	8 (8; 11)
Duration of treatment (days)	7 (3; 11)	6 (4; 11)	7 (6; 10)
Trough concentration (mg/L) ^a^	6.0 (3.5; 8.0)	6.0 (3.0; 9.0)	6.0 (3.3; 9.5)
Time from start of therapy (days)	2.0 (1.4; 2.7)	1.4 (1.0; 2.1)	2.0 (2.0; 2.1)
Time from start of infusion (h)	6.8 (5.9; 8.7)	10.9 (7.7; 11.0)	8.0 (8.0; 11.4)
Taken within ±1 h from end of dose interval	15 (79%)	8 (62%)	12 (86%)
Practical peak concentration (mg/L)	ND	ND	19 (15; 24)
Time from end of infusion (h)	ND	ND	2.0 (1.8; 2.0)

Data are presented as median (lower quartile; upper quartile) or n (% of treatments). ND = not determined. ^a^ In each group, three measured trough concentrations were below the limit of quantification (LOQ) (<2 mg/L with EMIT (n = 1) and <3 mg/L with KIMS (n = 8)) and they were set to one half of LOQ for pharmacokinetic calculations. Seven of nine below-LOQ concentrations occurred with a dose interval of 12 h.

**Table 3:** Model comparison using steady state trough concentrations (n = 28) and typical parameter values (a priori predictions) for combined Groups 2 and 3.

Model	Mean Percent Error (%)	Median Percent Error (%)	Root Mean Square Error (mg/L)	Predictions Within 15%	Additional Predictions Within 2.5 mg/L
Colin	+41%	+9%	3.73	8 (29%)	9 (32%)
Kloprogge	+80%	+43%	5.90	6 (21%)	7 (25%)
Le-1	+44%	+15%	4.56	9 (32%)	9 (32%)
Le-2	+72%	+41%	5.17	3 (11%)	7 (25%)
Le-3	+105%	+74%	6.25	3 (11%)	5 (18%)

**Table 4:** Bayesian parameter estimates for the initial dose regimen in Group 1 children from 0.3 to 12 years (n = 19) obtained with two best structural models and two error models using a single trough concentration (a posteriori predictions).

	Le-1 Model		Colin Model	
Parameter	Reduced Error Model	Original Error Model	Reduced Error Model	Original Error Model
Prediction error for trough concentration (mg/L)	0.0 (−0.3–+0.6)	+0.1 (−0.9–+1.9)	0.0 (−0.6–+0.8)	+0.1 (−2.2–+2.9)
Percent error for trough concentration (%)	+0.8 (−3.5–+7.7)	+2.6 (−12.6–+23.4)	+0.3 (−5.7–+9.6)	+1.5 (−24.6–+78.9)
AUC_24_ at steady state (mg·h/L)	303 (187–432)	314 (189–428)	294 (153–436)	278 (191–411)
Ratio of individual AUC_24_ to that by Le-1 reduced error model (%) ^a^	–	101 (96–109)	96 (77–124)	97 (76–127)
Clearance (CL) (mL/min/kg)	2.44 (1.57–3.44)	2.43 (1.63–3.40)	2.63 (1.56–4.20)	2.60 (1.75–3.38)
Vd or V1 (L/kg)	0.63 (0.59–0.67)	0.63 (0.60–0.67)	0.62 (0.54–0.75)	0.62 (0.57–0.72)
V2 (L/kg)	–	–	0.59 (0.38–0.78)	0.59 (0.48–0.71)
Q (mL/min/kg)	–	–	1.05 (0.62–1.65)	1.07 (0.74–1.59)
Distribution half-life (h)	–	–	1.7 (1.4–2.3)	1.7 (1.4–2.3)
Elimination half-life (h)	3.0 (2.0–4.8)	3.0 (2.1–4.6)	9.8 (7.7–13.8)	9.9 (8.8–13.6)

Data are presented as median (min–max). Structural models and error models are described in Supplementary Materials (Tables S1 and S2). Vd = Apparent volume of distribution (Le-1 is one-compartment model); V1 = Central volume of distribution; V2 = Peripheral volume of distribution; Q = Intercompartmental clearance. ^a^ Additionally, the ratio of individual AUC_24_ by Colin original to Colin reduced was 101 (87–125)%.

**Table 5:** Bayesian parameter estimates for the initial dose regimen in Group 2 children from 12.1 to 17 years (n = 13) obtained with two best structural models and two error models using a single trough concentration (a posteriori predictions).

	Le-1 Model		Colin Model	
Parameter	Reduced Error Model	Original Error Model	Reduced Error Model	Original Error Model
Prediction error for trough concentration (mg/L)	+0.1 (−0.9–+0.6)	+0.2 (−3.0–+1.9)	+0.1 (−1.1–+0.7)	+0.9 (−4.3–+2.3)
Percent error for trough concentration (%)	+2.3 (−7.7–+6.2)	+7.5 (−27.1–+18.7)	+3.6 (−10.2–+7.8)	+22.2 (−39.0–+111.5)
AUC_24_ at steady state (mg·h/L)	323 (183–471)	317 (187–487)	316 (151–557)	297 (212–543)
Ratio of individual AUC_24_ to that by Le-1 reduced error model (%) ^a^	–	102 (88–108)	95 (82–118)	104 (77–129)
Clearance (CL) (mL/min/kg)	1.99 (1.22–3.03)	1.96 (1.24–2.98)	2.02 (1.10–3.65)	1.83 (1.26–2.80)
Vd or V1 (L/kg)	0.61 (0.57–0.68)	0.62 (0.57–0.68)	0.65 (0.49–0.71)	0.63 (0.54–0.69)
V2 (L/kg)	–	–	0.58 (0.46–0.82)	0.60 (0.52–0.75)
Q (mL/min/kg)	–	–	0.82 (0.59–1.14)	0.85 (0.64–1.10)
Distribution half-life (h)	–	–	2.3 (1.7–3.2)	2.4 (1.9–3.0)
Elimination half-life (h)	3.6 (2.3–6.1)	3.7 (2.3–5.9)	13.1 (9.3–17.0)	13.8 (10.8–16.5)

Data are presented as median (min–max). Structural models and error models are described in Supplementary Materials (Tables S1 and S2). Vd = Apparent volume of distribution (Le-1 is one-compartment model); V1 = Central volume of distribution; V2 = Peripheral volume of distribution; Q = Intercompartmental clearance. ^a^ Additionally, the ratio of individual AUC_24_ by Colin original to Colin reduced was 112 (77–146)%.

**Table 6:** Vancomycin dosing and pharmacokinetic parameters for the initial dose regimen for different age groups.

Variable	Age 1–12 Months (n = 14)	Age 1–12 Years (n = 19)	Age 12.1–17 Years (n = 13)
Initial daily dose (mg/kg/day)	44 (42; 45)	41 (40; 46)	31 (30; 39)
Parameters: First-order equations/Le-1 reduced error	12/2	2/17	0/13
AUC_24_ at steady state (mg·h/L)	360 (253–640)	300 (187–432)	323 (183–471)
AUC_24_ 400–600 mg·h/L	4 (29%)	2 (11%)	2 (15%)
AUC_24_ 601–650 mg·h/L	2 (14%)	0	0
AUC_24_ 300–399 mg·h/L	3 (21%)	7 (37%)	5 (38%)
AUC_24_ <300 mg·h/L	5 (36%)	10 (59%)	6 (46%)
Clearance (CL) (mL/min/kg)	2.00 (1.02–2.85)	2.49 (1.57–3.44)	1.99 (1.22–3.03)
Dose to obtain median AUC_24_ of 400 mg·h/L (mg/kg/day)	48	60	48
Dose increases (n) ^a^	4 (29%)	7 (37%)	6 (46%)
Final change in dose (mg/kg/day)	10 (7; 14)	14 (11; 22)	17 (13; 23)
Final daily dose (mg/kg/day)	54 (52; 58)	55 (51; 65)	46 (41; 50)

Data are presented as median (lower quartile; upper quartile) or median (min–max) or n (% of treatments). The numbers of trough concentrations below the limit of quantification were 1, 5, and 3 in these groups, and they were set to one half of LOQ for pharmacokinetic calculations. ^a^ The final change in dose in relation to the initial dose and the final daily dose are calculated for these (n) treatments. Additionally, the daily dose was reduced during one treatment in the whole study.

## Data Availability

The original contributions presented in this study are included in the article. Additional data are not available based on the regulations of Kuopio University Hospital.

## Supplementary Materials

The following supporting information can be downloaded at: https://www.mdpi.com/article/10.3390/antibiotics15090852/s1, Table S1: Population pharmacokinetic models of vancomycin and conversion of creatinine values; Table S2: Bayesian forecasting with Le-1 and Colin models; Table S3: Validation of Bayesian parameter estimation; Table S4. Comparison of pharmacokinetic parameters obtained with Bayesian forecasting for Group 3 children from 1 to 15 months (n = 14) using steady-state equations; Table S5: Effect of repeated treatments and of concentrations below the lower limit of quantification (LOQ) on clearance and AUC_24_ estimates; Figure S1. Vancomycin concentration curves obtained with Le-1 reduced residual error model and Colin reduced residual error model for the median child in Group 1 (17 kg) (A,B) and in Group 2 (65 kg) (C,D) during a single dose interval at steady state (112–120 h) (A,C) and during the elimination phase (the last dose was given at 112 h) (B,D). Vancomycin dose was 15 mg/kg every 8th hour and the duration of infusion was 1 h (Group 1) or 2 h (Group 2). Median pharmacokinetic parameter values were taken from Table 4 and Table 5. There were no vancomycin concentrations from the late terminal elimination phase in our study, and it was impossible to resolve which model was more realistic for our patients.

## Abbreviations

The following abbreviations are used in this manuscript:

AUC_24_	Area under the curve over 24 h at steady state
CL	Clearance
CV%	Coefficient of variation
DEPS	Differential evolution and particle swarm optimization
EMIT	Enzyme-multiplied immunoassay technique
Enz	Enzymatic (assay)
ICU	Intensive care unit
IDMS	Isotope dilution mass spectrometry
IQR	Interquartile range
KIMS	Immunoassay based on kinetic interaction of microparticles in solution
LOQ	Lower limit of quantification
MIC	Minimum inhibitory concentration
MRSA	Methicillin-resistant *Staphylococcus aureus*
NICU	Neonatal intensive care unit
PCr	Plasma or serum creatinine level
PK	Pharmacokinetic
PMA	Postmenstrual age
RMSE	Root mean square error
TDM	Therapeutic drug monitoring
Vd	Apparent volume of distribution

## Appendix A

### IDMS-Traceable Enzymatic Plasma Creatinine for Bayesian Forecasting

IDMS-traceable enzymatic PCr for each patient in Groups 1 and 2 was defined and calculated for a priori predictions (n = 28) and a posteriori predictions (n = 32) (Section 4.4.2 and Section 4.4.3) based on the availability of PCr measurements in the following order of preference: (1) Mean value of pre-treatment PCr and PCr values measured during the treatment up to the time of vancomycin trough measurement (a priori n = 16; a posteriori n = 18); (2) Pre-treatment PCr alone (a priori n = 8; a posteriori n = 10); (3) PCr measured 1 to 4 days after the trough measurement (a priori n = 2; a posteriori n = 2); and (4) PCr was imputed using the equation for age-adjusted mean PCr [27] (a priori n = 2; a posteriori n = 2). The patients with imputed PCr (n = 2) showed no signs of kidney dysfunction, and the imputed values were close to the measured values in other patients of the same age.
