# Supplementary material for: Vancomycin AUC24 Estimation for Children with Bayesian Forecasting and First-Order Equations to Update Dose Recommendations
Source: Antibiotics (Basel). 2026 Sep 1;15(9):852. doi: 10.3390/antibiotics15090852 (PMC13603718; doi:10.3390/antibiotics15090852)
Supplement: Supplementary file 1 [file antibiotics-15-00852-s001.zip › antibiotics-4451389-supplementary.pdf]

Supplementary Materials

Table S1. Population pharmacokinetic models of vancomycin and conversion of creatinine values

Two-compartment model by Colin et al. 2019 [19]

$$\mathrm{CL\ (L/h)} = 5.31 \times \left[\frac{\mathrm{V1}}{42.9}\right]^{0.75} \times \left[\frac{PMA^{2.89}}{PMA^{2.89} + 46.4^{2.89}}\right] \times \left[\frac{\left(\frac{PMA}{52}\right)^{-2.24}}{\left(\frac{PMA}{52}\right)^{-2.24} + 61.6^{-2.24}} \times \frac{1}{0.771}\right] \times e^{-0.649 \times (SCR(mg/dL) - SCRstd)}$$

$$\mathrm{SCRstd\ (mg/dL)} = e^{\ln(SCRstd)}$$

$$\ln\mathrm{(SCRstd)} = -1.228 + log_{10}\left(\frac{PMA}{52}\right) \times 0.672 + 6.27 \times e^{\left(-3.11 \times \left(\frac{PMA}{52}\right)\right)}$$

$$\mathrm{V1\ (L)} = 42.9 \times \left[\frac{WT}{70}\right] \quad (= 0.613 \times \mathrm{WT})$$

$$\mathrm{V2\ (L)} = 41.7 \times \left[\frac{WT}{70}\right] \quad (= 0.596 \times \mathrm{WT})$$

$$\mathrm{Q\ (L/h)} = 3.22 \times \left[\frac{V2}{41.7}\right]^{0.75}$$

Two-compartment model by Kloprogge et al. 2019 [20]

$$\mathrm{CL}\ (L/h) = 4.84 \times \left[\frac{WT}{70}\right]^{0.632} \times \left[\frac{PMA^{3.52}}{PMA^{3.52} + 50.2^{3.52}}\right] \times \left[\frac{SCR_{Mean}}{SCR(\mu mol/L)}\right]^{0.692}$$

$$For\ age \le 15\ years\text{:} \quad SCR_{Mean}\ (\mu mol/L) = -2.37330 - 12.91367 \times \ln(Age) + 23.93581 \times \sqrt{Age}$$

For 15.1 – 17.9 $years$:

$$Boys\ SCR_{Mean}\ (\mu mol/L) = 9.5471 \times \mathrm{Age} - 87.847 \qquad Girls\ SCR_{Mean}\ (\mu mol/L) = 4.7137 \times \mathrm{Age} - 15.347$$

$$\mathrm{V1\ (L)} = 39.9 \times \left[\frac{WT}{70}\right] \quad (= 0.57 \times \mathrm{WT})$$

$$\mathrm{V2\ (L)} = 37.8 \times \left[\frac{WT}{70}\right] \quad (= 0.54 \times \mathrm{WT})$$

$$\mathrm{Q\ (L/h)} = 3.85 \times \left[\frac{WT}{70}\right]^{0.75}$$

One-compartment model by Le et al. 2013 [7]

$$\mathrm{CL}\ (L/h) = 0.248 \times WT^{0.75} \times \left[\frac{42.43}{SCR(\mu mol/L)}\right]^{0.361} \times \left[\frac{\ln(365 \times Age)}{7.8}\right]^{0.995}$$

$$\mathrm{Vd\ (L)} = 0.636 \times \mathrm{WT}$$

Conversions of creatinine values for Le model [21]

Enzymatic SCR to Jaffe SCR

$$\text{Jaffe SCR (mg/dL)} = \frac{Enzymatic\ SCR\ (mg/dL) + 0.122}{1.05}$$

Isotope dilution mass spectrometry (IDMS) traceable enzymatic SCR to Jaffe SCR

$$\text{Jaffe SCR (mg/dL)} = \frac{IDMS\ traceable\ enzymatic\ SCR\ (mg/dL) + 0.253}{1.027}$$

Pharmacokinetic parameters: CL = Clearance; Q = Intercompartmental clearance; V1 = Central volume of distribution; V2 = Peripheral volume of distribution; Vd = Apparent volume of distribution.
Covariates: Age = Age (years); PMA = Postmenstrual age (weeks); SCR = Serum or plasma creatinine (mg/dL in Colin model; µmol/L in Kloprogge and Le models; conversion: 1 mg/dL = 88.4 µmol/L); SCR_{Mean} = Age-adjusted mean SCR (µmol/L); SCRstd = Standardized SCR based on PMA (mg/dL); WT = Body weight (kg)

Table S2. Bayesian forecasting with Le-1 and Colin models

**Software and its settings**<br>Libre Office 7.4 (LibreOffice Community) and its Solver tool was used with Differential Evolution & Particle Swarm Optimization (DEPS) evolutionary algorithm. The default settings of DEPS algorithm were used except that variable bounds guessing was de-activated since the lower and upper boundaries of the estimated constants were set to 20% and 500% of the population values (see below).
**Equations and settings for Le-1 model [7]**<br>$\mathrm{OFV} = \frac{(C_{obs} - C_{pred})^2}{SD_{Cobs}{}^2} + \frac{(\ln(CL\_slope_{pop}) - \ln(CL\_slope_{est}))^2}{SD_{CL}{}^2} + \frac{(\ln(Vd\_slope_{pop}) - \ln(Vd\_slope_{est}))^2}{SD_{Vd}{}^2}$<br><br>Natural logarithm was used in CL and Vd terms since the unexplained between-subject variability was lognormal.<br>The terms in the numerator were squared to get squared residuals, and SD values were squared to get variances.<br><br>C_{obs} = Observed concentration<br>C_{pred} = Model-predicted concentration<br>CL_slope_{est} and Vd_slope_{est} replaced the population values in CL and Vd equations (Table 1S). The concentration obtained at the recorded time of trough measurement was calculated for each preceding dose using the recorded dose and time of dose, and these concentrations were summed to get C_{pred}. For Group 3, a second concentration term (the practical peak concentration) was added into OFV equation, and steady state pharmacokinetic equations were used.<br>CL_slope_{pop} = Population value of CL_slope (0.248 in Table S1)<br>CL_slope_{est} = Estimated individual value of CL_slope<br>Vd_slope_{pop} = Population value of Vd_slope (0.636 in Table S1)<br>Vd_slope_{est} = Estimated individual value of Vd_slope<br>SD_{Cobs} = SD for the residual error in observed concentration<br>Le-1 with reduced residual error: $SD_{Cobs} = 0.15 \times C_{obs}$<br>Le-1 with original residual error: $SD_{Cobs} = 0.29 \times C_{obs}$ [7]<br>SD_{CL} = SD for lognormal unexplained between-subject variability in CL (0.35) [7]<br>SD_{Vd} = SD for lognormal unexplained between-subject variability in Vd (0.18) [7]<br><br>The lower and upper boundaries for CL_slope_{est} and Vd_slope_{est} were 20% and 500% of the corresponding population values, and the boundaries were set in DEPS settings after natural log-conversion.
**Equations and settings for Colin model [19]**<br>OFV equation was similar to Le model, but there were parameter terms for CL, V1, and V2 (there is no unexplained between-subject variability in Q).<br>The population values for the slopes of CL, V1, and V2 are shown in Table S1.<br>C_{pred} was calculated similarly to Le-1 model. Notice that the individual estimate of V1 is included in CL equation and the individual estimate of V2 in Q equation (Table S1).<br><br>SD_{Cobs} = SD for the residual error in observed concentration<br>Colin with reduced residual error: $SD_{Cobs} = 0.15 \times C_{obs}$<br>Colin with original residual error: $SD_{Cobs} = 1.23 + 0.215 \times C_{obs}$ [19]<br>SD_{CL} = SD for lognormal unexplained between-subject variability in CL (0.273) [19]<br>SD_{V1} = SD for lognormal unexplained between-subject variability in V1 (0.268) [19]<br>SD_{V2} = SD for lognormal unexplained between-subject variability in V2 (0.827) [19]<br><br>The lower and upper boundaries for the parameters were defined in the same way as in Le model.
Statistical abbreviations: OFV = Objective Function Value; SD = standard deviation.<br>Pharmacokinetic parameters: CL = Clearance; Q = Intercompartmental clearance; V1 = Central volume of distribution; V2 = Peripheral volume of distribution; Vd = Apparent volume of distribution.

Table S3. Validation of Bayesian parameter estimation

	Estimates by Proost 1995 [22]				Estimates with Libre Office using DEPS algorithm			
**Run description**	**CL (L/h/kg)**	**Vd (L/kg)**	**C_{trough} (mg/L)**	**C_{peak} (mg/L)**	**CL (L/h/kg)**	**Vd (L/kg)**	**C_{trough} (mg/L)**	**C_{peak} (mg/L)**
Basic run	0.0356	0.440	12.0	21.7	0.0356	0.441	12.0	21.7
CL variability increased	0.0302	0.464	13.3	22.6	0.0302	0.464	13.3	22.6
Vd variability increased	0.0361	0.400	12.0	22.7	0.0360	0.401	12.0	22.7
Residual error increased	0.0420	0.471	10.3	19.3	0.0420	0.471	10.3	19.4

Proost 1995 [22] showed these theoretical runs on theophylline using one-compartment model and repeated bolus injections and used commercial Bayesian pharmacokinetic software for parameter estimation.
In our validation procedure, the same parameter estimates were obtained with Libre Office using DEPS algorithm.

Table S4. Comparison of pharmacokinetic parameters obtained with Bayesian forecasting for Group 3 children from 1 to 15 months (n=14) using steady-state equations

**A. Predictions with typical parameter values (a priori)**				
**Parameter**	**Le-1 model**		**Colin model**	
	**Trough**	**Peak**	**Trough**	**Peak**
Mean percent error (%)	+15%	-6%	+64%	-10%
Median percent error (%)	-12%	-4%	+36%	-9%
Root mean square error (mg/L)	4.96	6.09	4.99	7.49
Predictions within 15%	3 (21%)	6 (43%)	3 (21%)	5 (36%)
Additional predictions within 2.5 mg/L	4 (29%)	2 (14%)	3 (21%)	0
**B. Bayesian estimates (a posteriori)**				
	**Le-1 model**		**Colin model**	
**Parameter**	**Reduced error model**	**Original error model**	**Reduced error model**	**Original error model**
Prediction error for trough concentration (mg/L)	0.0 (-1.2 – +0.3)	-0.1 (-4.1 – +0.5)	+0.2 (-1.2 – +0.6)	+0.8 (-4.5 – +2.1)
Percent error for trough concentration (%)	-0.1 (-8.2 – +8.4)	-1.7 (-25.8 – +13.6)	+4.2 (-7.8 – +14.6)	+11.7 (-28.1 – +131.6)
Prediction error for peak concentration (mg/L)	-0.7 (-3.9 – +1.8)	-1.3 (-4.6 – +2.2)	-2.0 (-5.8 –+0.3)	-2.4 (-7.1 – +1.0)
Percent error for peak concentration (%)	-2.2 (-20.7 – +12.5)	-7.2 (-22.8 – +15.7)	-8.2 (-30.5 – +1.9)	-13.0 (-27.6 – +6.8)
AUC_{24} at steady state (mg·h/L)	330 (218–609)	332 (211–532)	316 (195–591)	326 (213–519)
Ratio of individual AUC_{24} to that by first-order equations (%)	97 (78–110)	94 (76–113)	93 (72–106)	92 (76–111)
Clearance (CL) (mL/min/kg)	2.34 (1.07–3.32)	2.30 (1.22–3.41)	2.41 (1.10–3.70)	2.32 (1.31–3.17)
Vd or V1 (L/kg)	0.64 (0.54–0.70)	0.64 (0.58–0.67)	0.56 (0.43–0.67)	0.61 (0.47–0.67)
V2 (L/kg)	–	–	0.48 (0.17–0.84)	0.56 (0.40–0.76)
Q (mL/min/kg)	–	–	1.29 (0.51–1.92)	1.42 (0.95–1.78)
Distribution half-life (h)	–	–	1.6 (1.4–1.9)	1.6 (1.4–1.9)
Elimination half-life (h)	3.3 (1.9–7.2)	3.2 (2.0–6.3)	8.2 (4.4–15.9)	8.8 (6.7–14.0)

Plasma creatinine values

IDMS-traceable enzymatic plasma creatinine (PCr) was defined for each patient based on available data: (1) Mean value of pre-treatment PCr and PCr values measured during the treatment up to the time of vancomycin trough measurement (n=3); (2) Pre-treatment PCr alone (n=8); (3) PCr measured during the course, but before the trough measurement (n=2); and (4) PCr was imputed using the equation for age-adjusted mean PCr [27] (n=1).

Comments

Overall, Bayesian AUC_{24} and CL estimates (a posteriori) were similar with all models, and close to those obtained with first-order equations (chapter 2.2.4). The accumulation half-life calculated with median parameters of Colin reduced model (AUC fraction of α-phase × Distribution half-life + AUC fraction of β-phase × Elimination half-life) was 5.0 h and the time interval from the start of vancomycin treatment to the trough measurement (measured before the peak) was ≥ 48 h in 13 of 14 treatments. Additionally, the concentration curve was calculated for the median patient with Colin reduced model and a dose of 15 mg/kg every 8th hour given as 1-hour infusion, and the peak concentration at 25 h (after five accumulation half-lives) was 98.0% of the final steady state value. These calculations indicated that steady-state equations were valid also for Colin model.

Data are presented as a single estimate, n (% of treatments) or median (min–max).
Vd = Apparent volume of distribution (Le-1 is one-compartment model); V1 = Central volume of distribution; V2 = Peripheral volume of distribution; Q = Intercompartmental clearance.

Table S5. Effect of repeated treatments and of concentrations below the lower limit of quantification (LOQ) on clearance and AUC_{24} estimates

<table>
<tr><td colspan="10">General information
AUC_{24} and CL estimates for different age groups were obtained with first order equations and Le-1 model with reduced residual error as described in Table 6.</td></tr>
<tr><td colspan="10">A. Repeated treatments
Change in parameter estimates (percent change compared with values in Table 6) when the same patient occurred only once in the whole study at his/her youngest age. The number of treatments (n) decreased in the youngest age group from 14 to 13, and in the middle age group from 19 to 16, whereas there was no change in the oldest age group.</td></tr>
<tr><td></td><td colspan="3">Age 1–12 months</td><td colspan="3">Age 1–12 years</td><td colspan="3">Age 12.1–17 years</td></tr>
<tr><td>Parameter</td><td>Median</td><td>Min</td><td>Max</td><td>Median</td><td>Min</td><td>Max</td><td>Median</td><td>Min</td><td>Max</td></tr>
<tr><td>CL (mL/min/kg)</td><td>+5%</td><td>+21%</td><td>same</td><td>+3%</td><td>same</td><td>same</td><td>–</td><td>–</td><td>–</td></tr>
<tr><td>AUC_{24} (mg×h/L)</td><td>-2%</td><td>same</td><td>-5%</td><td>-3%</td><td>same</td><td>same</td><td>–</td><td>–</td><td>–</td></tr>
<tr><td colspan="10">A. Below LOQ concentrations
Change in parameter estimates (percent change compared with values in Table 6) when all below LOQ concentrations (n=1, n=5, and n=3 from the youngest to the oldest age group) were set to LOQ (and not LOQ/2). This is an unlikely scenario.</td></tr>
<tr><td>CL (mL/min/kg)</td><td>same</td><td>same</td><td>+1%</td><td>-8%</td><td>same</td><td>-5%</td><td>-1%</td><td>same</td><td>-8%</td></tr>
<tr><td>AUC_{24} (mg×h/L)</td><td>same</td><td>-1%</td><td>same</td><td>same</td><td>+41%</td><td>same</td><td>same</td><td>+13%</td><td>same</td></tr>
<tr><td colspan="10">When below LOQ concentrations were set to LOQ/2 in all nine cases (the original calculations), AUC_{24} estimates were 183–273 mg×h/L. When below LOQ concentrations were set to LOQ in all nine cases, AUC_{24} estimates in eight cases were 227–296 mg×h/L and in one case exactly 300 mg×h/L. These calculations suggest that the correct AUC_{24} category in all nine cases was "< 300 mg×h/L". It is also possible that the true concentration was lower than LOQ/2, but no calculations were made for this scenario.</td></tr>
</table>

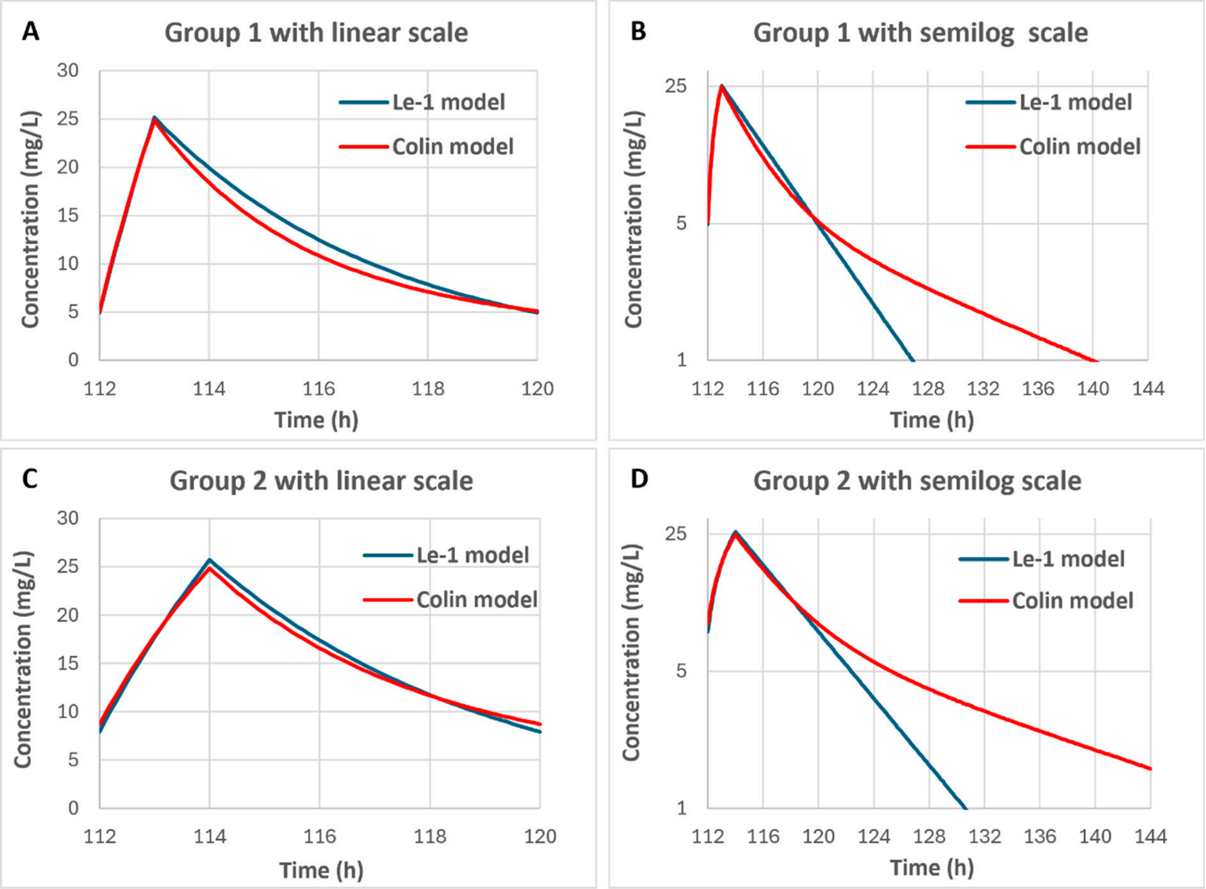

Figure S1. Vancomycin concentration curves obtained with Le-1 reduced residual error model and Colin reduced residual error model for the median child in Group 1 (17 kg) **(A, B)** and in Group 2 (65 kg) **(C, D)** during a single dose interval at steady state (112–120 h) **(A, C)** and during the elimination phase (the last dose was given at 112 h) **(B, D)**. Vancomycin dose was 15 mg/kg every 8th hour and the duration of infusion was 1 h (Group 1) or 2 h (Group 2). Median pharmacokinetic parameter values were taken from Tables 4 and 5. There were no vancomycin concentrations from the late terminal elimination phase in our study, and it was impossible to resolve which model was more realistic for our patients.
